# Social Skills and Creativity as Elements That Enhance Resilience in Adolescence

**DOI:** 10.3390/bs14121158

**Published:** 2024-12-02

**Authors:** Alba González Moreno, María del Mar Molero Jurado

**Affiliations:** Department of Psychology, University of Almería, 04120 Almería, Spain; mmj130@ual.es

**Keywords:** resilience, social skills, creativity, adolescents

## Abstract

Resilience plays a crucial role in overcoming the adversities and challenges faced by young people during adolescence. Current research focuses on understanding the factors that contribute to the development of resilience at this stage, with the goal of promoting the well-being and mental health of young people. In this descriptive cross-sectional study, we examined the relationship between social skills, creativity and resilience in a sample of 743 students aged 14 to 19 years from different educational centers in the province of Almería (Spain). We used the Social Skills Questionnaire (CHASO), the Turtle Creativity Questionnaire and the reduced resilience scale CDRISC-10 to collect data. Data analysis was performed using correlation analysis, Student’s *t*-test for differences according to sex, analysis of variance to test for differences according to resilience groups, multiple regression analysis for predictors of resilience and simple mediation analysis. The results showed a positive and significant relationship between social skills, creativity, and resilience. The differences found suggest that being male and having a higher level of resilience increases the likelihood of having higher social skills and resilience. Social skills and creativity predicted resilience and social skills mediated the relationship between creativity and social skills. These findings highlight the importance of strengthening social skills and promoting creativity to enhance resilience in adolescents, which may have practical implications for the design of intervention and support programs.

## 1. Introduction

Adolescence is a critical stage in human development, characterized by significant physical, cognitive, emotional and social changes [1]. During this period, adolescents face a number of challenges and adversities that can have a profound impact on their psychological well-being and ability to cope with life’s difficulties [2,3,4,5]. However, many adolescents demonstrate a remarkable ability to adapt and recover from stressful situations, which is attributed to their resilience [6].

Resilience is defined as a person’s capacity to face and overcome adversities, developing internal and external skills and resources that allow them to recover and grow from these challenging experiences [7]. One of the most characteristic aspects of resilience is the individual’s ability to recover and return to their original functioning after a stressful event, so the current focus is on strengthening resilience in the face of traumatic situations [8]. Resilience develops when people learn to adapt constantly in their daily lives to different contexts and challenges, not only by reacting to crises or risks, but also as a continuous process that involves adjusting actions and strategies to successfully face the changing demands of the environment [9]. In the context of adolescence, resilience becomes especially relevant due to the complexity of changes and transitions that adolescents face, such as increased family responsibilities, higher academic and social demands, separation from family and exploration of new stressful experiences with peers and adult activities [10]. The diversity of challenges and events that emerge in this period reinforces the growing understanding of the importance of adolescence as a critical stage for the reorganization of the individual’s regulatory system that is filled with both opportunities and risks [11]. Resilience in adolescence is estimated to act as a protective factor because youth with higher resilience have a lower risk of depression compared to those with lower resilience [12]. Recent studies indicate that youth with lower resilience are more likely to experience more intense symptoms of depression and anxiety, as well as even more severe suicidal thoughts [13]. In contrast, a high degree of resilience acts as a protective factor against various mental health conditions, as it can significantly predict a reduction in anxiety and depression [14,15]. This idea is linked to the fact that the degree of depression of young people has both direct and indirect effects on the mental resilience of these subjects [16], thus studies verify that resilience acts as a moderator in the relationship between depression and risk factors such as child abuse [17]. A high level of resilience has been associated with an increase in positive experiences, as well as greater personal well-being and a decrease in psychological distress [18]. Thus, it is established that more resilient adolescents tend to manifest more positive behaviors and face fewer mental health problems [19]. Based on gender differences, boys were found to show higher resilience scores than girls [20].

In recent years, increased attention has been paid to understanding the factors that contribute to the development of resilience in adolescence, with the goal of promoting the well-being and mental health of young people [21,22,23]. Two elements that have received considerable attention in the literature are social skills and creativity.

Social skills refer to the set of competencies that enable adolescents to interact and relate effectively with others [24]. These skills include the ability to establish healthy relationships, communicate effectively, resolve conflicts constructively and show empathy toward others [25]. Well-developed social skills were shown to contribute to adolescent resilience by enabling them to establish and maintain social support networks, cope with difficult situations and overcome obstacles in their environment [26]. Previous studies expose that resilience helps to reduce the effects of stress, and overcoming adversity and is related to higher life skills and lower emotional and behavioral problems [27]. In addition, it was found that adolescents who experience greater development in social skills and emotional learning also experience a greater increase in resilience, prosocial behavior and a decrease in difficulties [28]. Regarding gender differences, it is estimated that boys score higher in social skills in general and in certain dimensions such as public speaking or coping with situations of ridicule, while girls score higher in social skills related to expressing positive feelings or apologizing [29].

On the other hand, creativity was identified as a key factor in the development of resilience in adolescence. Creativity is a fundamental factor in promoting social harmony, sustainable human development, technological innovation and scientific revolution, and although there is no unanimous concept about it, it is understood as the ability to generate original ideas and novel solutions to problems [30]. Therefore, it can be an optimal resource to face the various challenges of our society and manage the changes that people have to cope with [31]. In the context of resilience, creativity is considered a valuable tool, as it fosters cognitive flexibility and the ability to find new ways to face challenges [32]. Studies have shown that creativity fosters positive emotions and reduces perceived stress [33]. In addition, it is estimated that there is a positive relationship between creativity and resilience [34]. In this regard, recent research has shown that a moderate level of academic stress, when perceived as challenging, can enhance adolescents’ self-rated creativity, especially when resilience and academic self-efficacy act as mediating factors in this process [35]. Creativity is a key factor in promoting resilience, as resilient and creative people share qualities such as flexibility, resourcefulness, and adaptability [36,37]. Therefore, adolescents with higher levels of creativity also have higher levels of resilience, which allows them to have a greater ability to adapt to stressful situations and find innovative solutions to problems [38]. Regarding gender differences, the previous literature shows that there are no significant differences that allow for distinguishing this ability among adolescents [39].

### Aim of the Study and Hypotheses

The aim of this research is to identify how social skills and creativity are related to resilience in the adolescent stage. The initial hypotheses considered are the following:

**H1:**  *Social skills and creativity are positively correlated with resilience.*

**H2:**  *There are differences according to the sex of the students in social skills, creativity and resilience.*

**H3:**  *Different resilience groups score differently on social skills and creativity.*

**H4:**  *Social skills and creativity act as predictors of resilience.*

**H5:**  *Social skills mediate the relationship between creativity and resilience.*

## 2. Materials and Methods

### 2.1. Study Design and Participants

A cross-sectional descriptive approach was used in this quantitative study, following the guidelines of the STROBE statement for studies of this type [40].

The study sample consisted of 743 secondary school students, of whom 377 (50.7%) were girls and 366 (49.3%) were boys. The age of the participants ranged from 14 to 19 years (*M* = 14.99; *SD* = 0.86). All were students enrolled in different educational centers in the province of Almería, Spain. The academic years were mainly third (50.7%) and fourth (49.1%) years of secondary education. The great majority of the students were of Spanish nationality (92.9%), although students of other nationalities, such as Colombia, Morocco, Russia and Argentina were also included.

### 2.2. Instruments

The evaluation of the students was carried out using a set of instruments validated by other researchers in Spanish adolescents. In addition to these instruments, an ad hoc questionnaire was included that collected sociodemographic information on the adolescents. The instruments used in the study were the following:

- Social Skills: The Social Skills Questionnaire (CHASO) [41] was used to analyze the social skills of the participants. This questionnaire, validated in Spanish adolescents, consisted of 40 items distributed in 10 dimensions. Responses were rated on a five-point Likert scale (1 = Very uncharacteristic of me; 2 = Not very characteristic of me; 3 = Moderately characteristic of me; 4 = Fairly characteristic of me; 5 = Very characteristic of me). The reliability obtained by means of this questionnaire was good in each of its dimensions: (1) Interacting with strangers (Skill 1; *α* = 0.75), (2) Expressing positive feelings (Skill 2; *α* = 0.77), (3) Coping with criticism (Skill 3; *α* = 0.71), (4) Interacting with people I am attracted to (Skill 4; *α* = 0.88), (5) Remaining calm in the face of criticism (Skill 5; *α* = 0.59), (6) Public speaking/interacting with superiors (Skill 6; *α* = 0.71), (7) Coping with situations of making a fool of oneself (Skill 7; *α* = 0.59), (8) Defending one’s rights (Skill 8; *α* = 0.67), (9) Apologizing (Skill 9; *α* = 0.81), (10) Refusing requests (Skill 10; *α* = 0.71). The total score on social skills also obtained high reliability indices (*α* = 0.86).
- Creativity: Students’ creativity was assessed using the Turtle Creativity Questionnaire [42], which consisted of a total score based on 31 items with dichotomous responses. This questionnaire assesses creativity in a general youths’ way according to the answers obtained. To do so, participants had to indicate whether or not they identified with each of the items (e.g., very imaginative storytelling or ingenious problem solving). An acceptable internal consistency was obtained (*α* = 0.65).
- Resilience: Youth resilience was assessed using the reduced variant CD-RISC10 [43], based on the original Connor–Davidson (CD-RISC) scale [44]. This scale consisted of ten items that were scored on a Likert scale with four response options. The responses measure the human ability to cope with traumatic situations (e.g., I am able to adapt when changes arise). Internal consistency was good (*α* = 0.83).

### 2.3. Procedure

For data collection, several secondary schools in the province of Almería (Spain) were contacted. Six public high schools agreed to participate in the study, and a date was agreed with the school authorities to carry out the data collection. The entire educational community, including teachers, families and students, received information about the objective of the study, its anonymous and voluntary nature. Once the consent of the participants and their legal guardians had been obtained, data collection proceeded between February and June 2022. This study was approved by the Bioethics Committee on Human Research of the University of Almeria, whose reference is UALBIO2021/025.

### 2.4. Data Analysis

The data collected were recorded in the statistical program SPSS version 28 [45] for subsequent analysis. Cronbach’s alpha coefficient was used to calculate the reliability of the instruments used. The results of the coefficient were interpreted according to the categories proposed by Cronbach [46] (<0.5 unacceptable, >0.5 poor, >0.6 questionable, >0.7 acceptable, >0.8 good and >0.9 excellent).

A descriptive analysis was carried out to provide detailed information on the adolescents who participated in the study. Likewise, a bivariate correlation analysis was performed using Pearson’s coefficient, with the purpose of identifying possible significant relationships between the variables evaluated. The results obtained were interpreted according to the categories established by Pearson [47] to measure the magnitude of the correlation: no correlation (values between 0 and 0.10), weak correlation (between 0.10 and 0.29), moderate correlation (between 0.30 and 0.50) and strong correlation (between 0.50 and 1.00).

Student’s *t*-test for independent samples was used to investigate the differences between sexes in relation to the variables examined in adolescent students. In addition, the effect size was calculated using Cohen’s d [48] to interpret the results of said test, which is interpreted by the following scores: <0.50 small; 0.50–0.80 medium; and ≥0.80. On the other hand, to know the differences according to the resilience groups (g1 = low resilience; g2 = medium resilience; and g3 = high resilience) in comparison with the different dimensions of social skills and creativity, an analysis of variance (ANOVA) was performed. To know the effect size in such analysis eta squared (*η*^2^) was used, which is usually considered around 0.01 to be a small effect, around 0.06 indicates a moderate effect and above 0.14 is considered a large effect [49].

A stepwise multiple regression analysis was conducted, using resilience as the dependent variable, with the aim of exploring the association of this variable with the predictors (social skills and creativity). Change statistics, regression coefficients and collinearity were taken into account to understand changes in effect sizes. A statistical significance level of *p <* 0.05 was established.

A simple mediation analysis was conducted to examine the role of social skills as a mediating variable in the relationship between creativity and resilience. This analysis was performed using the *medmod* module integrated into the jamovi v.2.3.2 software [50], which allows the calculation of mediation models and provides data such as standardized coefficients, *z* scores and significance levels. The *bootstrap* technique was applied with estimates based on 5000 samples, establishing a 95% confidence interval.

## 3. Results

### 3.1. Descriptive Analyses and Correlations

The correlation analysis revealed significant results that point to the existence of positive correlations between the three variables analyzed: social skills, creativity and resilience.

As shown in Table 1, a significant positive correlation was found between social skills and resilience (*r* = 0.36; *p* < 0.001) and creativity and resilience (*r* = 0.32; *p* < 0.001). Furthermore, such an association is also shown between social skills and creativity (*r* = 0.44; *p* < 0.001).

### 3.2. Differences Found in the Variables Examined According to Sex

Table 2 presents the results obtained on the differences according to sex in the different dimensions of social skills, creativity and resilience. The data reveal significant disparities between boys and girls in terms of the scores obtained in different aspects of social skills and resilience.

Regarding social skills, it was observed that boys scored higher in the following dimensions: interacting with people they are attracted to, keeping calm in the face of criticism, public speaking/interacting with superiors, coping with situations of making a fool of oneself, refusing requests and the total social skills score. On the other hand, girls showed outstanding performance in the dimension of expressing positive feelings and coping with criticism.

In relation to resilience, it was observed that boys obtained higher scores compared to girls.

On the other hand, no significant differences were found between boys and girls in the creativity dimension.

### 3.3. Differences Found According to the Resilience Groups in the Examined Variables

Table 3 shows the differences found according to the different resilience groups (low, medium and high) and social skills and creativity.

Analysis of variance (ANOVA) revealed that adolescents with lower levels of resilience (g1) obtain significantly lower scores in several dimensions of social skills, such as interacting with strangers (*F* = 10.99; *p <* 0.001; *η*^2^ = 0.02), coping with criticism (*F* = 8.07; *p <* 0.001; *η*^2^ = 0.05), interacting with people I am attracted to (*F* = 8.20; *p <* 0.001; *η*^2^ = 0.02), keeping calm in the face of criticism (*F* = 32.12; *p <* 0.001; *η*^2^ = 0.08), speaking in public/interacting with superiors (*F* = 52.04; *p <* 0.001; *η*^2^ = 0.12), standing up for one’s rights (*F* = 17.05; *p <* 0.001; *η*^2^ = 0.04), refusing requests (*F* = 13.86; *p <* 0.001; *η*^2^ = 0.03) and in the total social skills score (*F* = 54.71; *p <* 0.001; *η*^2^ = 0.12). In addition, this same group with lower resilience also showed lower levels of creativity compared to their medium and high resilience peers (*F* = 22.62; *p <* 0.001; *η*^2^ = 0.07).

However, the group of medium resilient adolescents (g2) obtained higher scores in the dimensions of expressing positive feelings (*F* = 8.07; *p <* 0.001; *η*^2^ = 0.02), coping with situations of making a fool of oneself (*F* = 5.16; *p <* 0.006; *η*^2^ = 0.01) and apologizing (*F* = 4.04; *p <* 0.018; *η*^2^ = 0.01) compared to their other low resilience peers.

### 3.4. Predictive Value of Social Skills and Creativity on Resilience

A stepwise multiple linear regression analysis was carried out to examine the relationship between resilience and other investigated variables such as social skills and creativity. Table 4 shows the associations found in the multiple regression analysis between the predictor variables and the dependent variable (resilience), indicating the standardized effect sizes (*β*) and the variance explained (*R-squared*). This linear regression analysis consists of two predictor models for adolescent resilience. Model I explains 37% of the variance and includes the dependent variable and social skills as predictors. On the other hand, Model II explains 41% of the variance and includes the dependent variable, social skills and creativity as predictors.

Model II offers the greatest explanatory capacity (*R*^2^ = 0.17) by including all predictors. It is observed that the most influential predictor in this model is social skills, although both social skills and creativity present a statistically significant impact (*p* < 0.001). Finally, the results of the statistics on the tolerance values and the variance inflation factor (VIF) show an absence of collinearity.

### 3.5. The Mediating Role of Social Skills in the Relationship Between Creativity and Resilience

**Figure 1:**
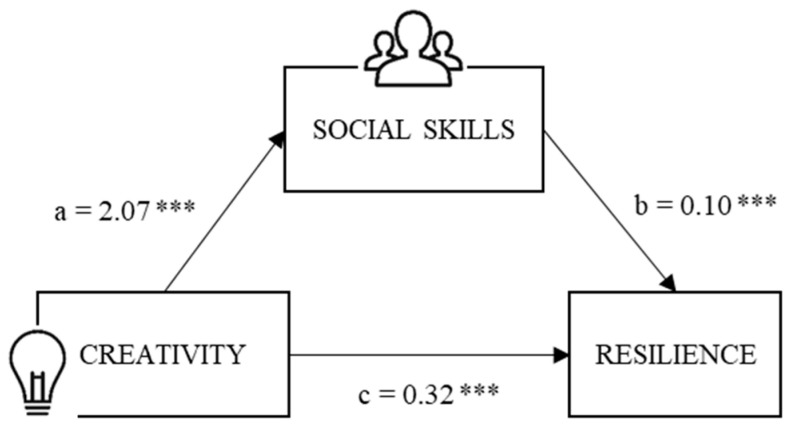
Path estimates. Note. *** *p* < 0.001.

Based on the results obtained, our objective was to assess whether social skills can act as a mediating variable in the relationship established between creativity and resilience in adolescents. To this end, a mediation model was computed, taking creativity (X) as an independent variable, the global score in social skills as a mediator (M) and, finally, resilience as a dependent variable (Y) (Figure 1).

The estimation of direct effects (X → Y) revealed a significant impact of creativity on resilience, with *β* = 0.323, *SE* = 0.071, 95% *CI* (0.181, 0.465), *Z* = 4.56, *p* < 0.001. Regarding indirect effects (X → M → Y), significance was found in the interactions between the variables in the model, with *β* = 0.21, *SE* = 0.032, 95% *CI* (0.154, 0.281), *Z* = 6.59, *p* < 0.001, indicating a mediation percentage of 39% (Table 5).

## 4. Discussion

This research has allowed us to verify how social skills and creativity are two variables that are related to resilience in adolescence. We wanted to investigate resilience because it refers to the ability of human beings to face complex situations [6,7,8]. This ability is of great importance in adolescence because it is considered a stage of changes and challenges [1,2,3,4,5,10]. This period of life, characterized by the search for identity, independence and skills to manage interpersonal relationships, underscores the need to understand how other capacities such as social skills and creativity can contribute to fostering adolescent resilience and well-being [21,22,23].

The results obtained in this study point to a significant and positive correlation of resilience with both social skills and creativity. These results relate to hypothesis 1 of the present study, which indicated that social skills and creativity are positively correlated with resilience. It is noteworthy how social skills enable positive social relationships, while creativity enables conflict resolution [25,30]. This positive relationship is also exposed in previous studies, as social skills and creativity are estimated to contribute to the development of resilience [26,34]. From a theoretical perspective, social skills enhance adolescents’ ability to establish affective bonds and support networks, which are essential for overcoming adversity. At the same time, creativity fosters a flexible and adaptive mindset, enabling adolescents to deal effectively with challenges.

An important aspect of this study is the analysis of sex differences in levels of resilience, social skills and creativity. The results show that boys tend to have higher levels of resilience and social skills compared to girls, which is consistent with previous research that has documented similar differences [20,29]. One possible explanation for this finding lies in gender roles and social expectations that, in many cultures, encourage skills such as assertiveness and independence in boys, while girls may face greater barriers to developing these competencies. However, no significant differences were found between boys and girls in creativity, which is supported by systematic reviews indicating that this ability is not significantly influenced by sex [39]. This suggests that creativity might be a more intrinsic and universal ability, less affected by cultural or socialization factors, compared to social skills and resilience. After these results, we can argue that hypothesis 2 of the study has not been fully accepted. Although sex differences were found between social skills and resilience, no such differences were obtained for creativity.

In addition to the differences according to sex, we also wanted to investigate the differences according to the resilience group. These groups were established as low, medium and high resilience according to the scores obtained. The data analyzed indicate that young people with lower mean scores in resilience have lower scores in social skills and creativity. These results verify hypothesis 3 of the study since differences were found according to the resilience group in the level of creativity and social skills of our participants. This evidence may be due to the fact that resilient youth possess higher capacities for problem solving, adapting to change, life skills and emotional learning [12,27,38]. It is noteworthy that young people with high resilience tend to possess greater abilities to face daily challenges, which could be related to previous experiences that have strengthened their ability to handle stress. This underscores the importance of implementing programs aimed at adolescents with low resilience, fostering the development of social skills and creativity as tools to improve their overall well-being.

Based on the significant relationships between social skills, creativity and resilience, the aim was to determine whether social skills and creativity predict resilience. The regression model indicates that social skills and creativity have the ability to predict or influence levels of resilience. This means that youth with more developed social skills and greater creativity may be more likely to have greater resilience. Based on this evidence, we can affirm that hypothesis 4 of the study, which indicates that social skills and creativity act as predictors of resilience, is fulfilled. Said results attend to the fact that adolescents with high levels of social skills tend to present fewer social difficulties and creativity helps to cope with challenges and reduce stress, which is consistent with previous research [32,33]. The predictive role of these skills is better understood when considering that social skills enable adolescents to navigate challenging social contexts more effectively, while creativity provides them with tools to approach problems in innovative and adaptive ways.

Finally, a mediation model was carried out to examine the mediating role of social skills in the relationship between creativity and resilience, being able to establish the interaction of these variables in the model. Thus, hypothesis 5 of the present study is established, which states that young people’s social skills mediate the relationship between creativity and resilience. This finding, in line with previous studies [28], highlights the fact that youth who increase their level of social skills in turn experience an increase in their level of resilience. In practical terms, this means that youth development programs should integrate activities that simultaneously promote creativity and social skills, as together they can amplify their positive impact on resilience.

## 5. Conclusions

In conclusion, it is worth noting how this study has clearly and convincingly demonstrated that there are significant relationships between social skills, creativity and resilience. The findings obtained not only underscore the intrinsic connection between these variables but also reinforce the idea that social skills and creativity play a crucial role in the development and strengthening of resilience. This has important implications, as it suggests that fostering these competencies may be an effective strategy to improve adolescents’ ability to cope with adverse situations, promoting their emotional and social adaptation more effectively.

From a practical perspective, the results of this study offer valuable input for the design and implementation of educational and intervention programs. These programs should prioritize the inclusion of activities oriented to the development of social skills and the stimulation of creativity as essential components. For example, group dynamics, problem-solving workshops and artistic activities could be implemented to allow adolescents to explore and express their ideas while strengthening their ability to interact and collaborate with others. This would contribute not only to the development of resilience but also to the strengthening of other important skills such as empathy, effective communication and critical thinking.

Furthermore, this approach has great potential for application in mental health contexts, especially in interventions designed to improve the emotional well-being and adaptive capacity of adolescents in vulnerable situations. The integration of strategies that enhance creativity and social skills could facilitate emotional recovery, improve self-esteem and promote a more positive outlook in the face of the challenges of everyday life. In this sense, strengthening resilience through the development of these competencies could translate into a significant improvement in the quality of life of adolescents, both in the present and in their future personal and social development.

In short, the results obtained in this study not only highlight the importance of social skills and creativity as pillars for the development of resilience but also open the door to multiple possibilities for intervention in educational, social and mental health settings. These findings invite further research to optimize strategies and programs that make the most of the potential of these skills in strengthening resilience, with the goal of improving the ability of adolescents to face and overcome the challenges that life presents them.

### Limitations and Future Research

This study has several limitations that should be considered when interpreting its findings. One of the main limitations is the scarcity of previous literature that jointly analyzes the three constructs: social skills, creativity and resilience. This lack of background makes it difficult to place the results in a consolidated theoretical framework and limits comparison with other studies. In addition, the cross-sectional design of the study represents a barrier to establishing causal relationships between the variables analyzed, since it only allows us to observe associations at a specific moment in time, without detailing how these relationships may evolve over time.

Likewise, the sample used may not be completely representative of the general population of adolescents, which limits the generalizability of the results to other cultural, social or educational contexts. Factors such as ethnic diversity, socioeconomic status, and individual differences in access to support resources may significantly influence the interaction between social skills, creativity, and resilience, and their impact has not been fully explored in this study.

As for future lines of research, longitudinal studies are highly recommended to analyze how these variables influence each other over time and to what extent their relationship may be mediated or moderated by other factors. Also, experimental designs could provide stronger evidence on the direction of the effect between social skills, creativity, and resilience. For example, specific interventions that strengthen these skills could be developed and their impact evaluated through rigorous methodologies that include control groups and long-term follow-up. It would also be interesting to explore other contextual and personal variables that could influence the relationship between the three competencies. These variables could include factors such as family support, the quality of interpersonal relationships, exposure to stressful situations, and differences in the cognitive or emotional abilities of adolescents. Similarly, one could delve deeper into how these relationships vary by sex, age, or cultural context. Another promising area for future research would be to examine the efficacy of practical interventions specifically designed to improve social skills and stimulate creativity, with the aim of strengthening resilience. Studies evaluating the impact of educational programs, creative workshops or therapeutic strategies in school and community settings could provide valuable information for the design of effective public policies and support programs. In addition, it would be important to measure not only the immediate effects of these interventions but also their long-term sustainability.

## Figures and Tables

**Table 1:** Descriptive and correlation matrix between social skills, creativity and resilience (*N* = 743).

	Social Skills	Creativity	Resilience
Social Skills	-		
Creativity	0.44 ***	-	
Resilience	0.36 ***	0.32 ***	-
Mean	126.62	17.65	26.65
SD	20.23	4.27	7.00
Min.	66	1	5
Max.	186	29	40

*** *p* < 0.001.

**Table 2:** Descriptive data and *t*-test according to sex (girls *n* = 377; boys *n* = 366).

		Sex				*t*	*p*	*d*
		Boys		Girls				
		*Mean*	*SD*	*Mean*	*SD*			
Social Skills	Interacting with strangers	10.50	3.62	9.96	3.97	1.91	0.056	-
	Expressing positive feelings	14.01	3.98	**15.43**	3.92	−4.87 ***	<0.001	0.36
	Dealing with criticism	14.71	3.45	**15.39**	3.50	−2.68 **	0.007	0.20
	Interacting with people I am attracted to	**10.13**	4.87	8.12	4.34	5.92 ***	<0.001	0.44
	Keeping calm in the face of criticism	12.62	3.50	12.03	3.18	2.38 *	0.017	0.17
	Public Speaking/Interacting with Superiors	**12.49**	3.67	10.88	3.81	5.84 ***	<0.001	0.43
	Dealing with situations of making a fool of oneself	**11.12**	3.47	10.58	3.35	2.14 *	0.032	0.16
	Defending one’s rights	13.10	3.78	12.85	3.95	0.87	0.383	-
	Apologize	15.42	3.74	15.79	3.66	−1.37	0.168	-
	Rejecting requests	**14.38**	3.55	13.79	3.72	2.18 *	0.029	0.16
	Total SS.SS	**128.46**	20.89	124.83	19.44	2.45 *	0.014	0.18
Creativity	Total Creativity	17.57	4.52	17.72	4.01	−0.41	0.682	-
Resilience	Total Resilience	**27.34**	7.13	25.99	6.81	2.64 **	0.008	0.19

* *p* < 0.05; ** *p* < 0.01; *** *p* < 0.001.

**Table 3:** Differences according to different resilience groups.

Scale		Resilience Groups	*N*	*Mean*	*SD*	ANOVA		Difference in Averages
						*F*	Sig.	
SS.SS	Interacting with strangers	Low (g1)	395	9.63	3.61	10.99	<0.001	|g1–g2| *** |g2–g3| |g1–g3| *
		Mean (g2)	282	10.85	3.89			
		High (g3)	66	**11.18**	4.02			
	Expressing positive feelings	Low (g1)	395	14.18	4.09	8.07	<0.001	|g1–g2| *** |g2–g3| |g1–g3|
		Mean (g2)	282	**15.38**	3.80			
		High (g3)	66	15.23	4.00			
	Dealing with criticism	Low (g1)	395	14.28	3.59	22.74	<0.001	|g1–g2| *** |g2–g3| |g1–g3| ***
		Mean (g2)	282	15.84	3.15			
		High (g3)	66	**16.35**	3.13			
	Interacting with people I am attracted to	Low (g1)	395	8.51	4.38	8.20	<0.001	|g1–g2| ** |g2–g3| |g1–g3| *
		Mean (g2)	282	9.60	4.84			
		High (g3)	66	**10.61**	5.55			
	Keeping calm in the face of criticism	Low (g1)	395	11.45	3.16	32.12	<0.001	|g1–g2| *** |g2–g3| |g1–g3| ***
		Mean (g2)	282	13.17	3.22			
		High (g3)	66	**13.91**	3.55			
	Public Speaking/Interacting with Superiors	Low (g1)	395	10.43	3.45	52.04	<0.001	|g1–g2| *** |g2–g3| |g1–g3| ***
		Mean (g2)	282	12.92	3.63			
		High (g3)	66	**13.76**	4.16			
	Dealing with situations of making a fool of oneself	Low (g1)	395	10.47	3.27	5.16	0.006	|g1–g2| * |g2–g3| |g1–g3|
		Mean (g2)	282	**11.23**	3.50			
		High (g3)	66	11.42	3.70			
	Defending one’s rights	Low (g1)	395	12.31	3.72	17.05	<0.001	|g1–g2| *** |g2–g3| * |g1–g3| ***
		Mean (g2)	282	13.45	3.88			
		High (g3)	66	**14.94**	3.75			
	Apologize	Low (g1)	395	15.25	3.77	4.04	0.018	|g1–g2| * |g2–g3| |g1–g3|
		Mean (g2)	282	16.04	3.48			
		High (g3)	66	15.92	4.01			
	Rejecting requests	Low (g1)	395	13.44	3.63	13.86	<0.001	|g1–g2| *** |g2–g3| |g1–g3| ***
		Mean (g2)	282	14.71	3.51			
		High (g3)	66	**15.22**	3.60			
	Total SS.SS	Low (g1)	395	119.94	19.14	54.71	<0.001	|g1–g2| *** |g2–g3| |g1–g3| ***
		Mean (g2)	282	133.19	18.28			
		High (g3)	66	**138.53**	20.09			
Creativity	Total Creativity	Low (g1)	287	16.53	4.38	22.62	<0.001	|g1–g2| *** |g2–g3| |g1–g3| ***
		Mean (g2)	232	18.57	3.84			
		High (g3)	51	**19.71**	3.79			

* *p* < 0.05; ** *p* < 0.01; *** *p* < 0.001.

**Table 4:** Stepwise multiple linear regression model.

Models	*R*	*R* ^2^	*R*^2^ Corrected	Statistics of Change				Durbin Watson	
				Standard Error of Estimation	Change in *R*^2^	Change in *F*	Sig. of Change in *F*		
**1**	0.37	0.14	0.14	6.40	0.14	95.24	<0.001		
**2**	0.41	0.17	0.17	6.28	0.03	22.24	<0.001	1.53	
**Models**			**Unstandardized coefficients**		**Standardized coefficients**	***t***	**Sig.**	**Collinearity**	
			***B***	**Typical error.**	**Beta**			**Tol.**	**VIF**
**1**	(Constant)		10.44	1.71		6.07	<0.001		
	Social Skills		0.13	0.01	0.37	9.75	<0.001	1.00	1.00
**2**	(Constant)		8.58	1.73		4.95	<0.001		
	Social Skills		0.10	0.01	0.29	6.85	<0.001	0.80	1.23
	Creativity		0.32	0.06	0.20	4.71	<0.001	0.80	1.23

**Table 5:** Mediation estimates.

Effect	Label	Estimate	*SE*	95% *CI*		*Z*	*p*	Mediation
				Lower	Upper			
Indirect	a × b	0.207	0.0345	0.140	0.276	5.99	<0.001	39
Direct	c	0.323	0.0710	0.181	0.465	4.56	<0.001	61
Total	c + a × b	0.530	0.0675	0.395	0.660	7.86	<0.001	100

## Data Availability

The data are available upon request from the corresponding author.
